# “It Was a Mistake, but We Knew That Something Might Happen”: Narratives of Teenage Girls' Experiences With Unintended Teenage Pregnancy

**DOI:** 10.3389/frph.2021.639544

**Published:** 2021-11-05

**Authors:** Busisiwe Nkala-Dlamini

**Affiliations:** Department of Social Work, School of Human and Community Development, University of the Witwatersrand, Johannesburg, South Africa

**Keywords:** unintended teenage pregnancy, social-sexual relationship, sexual risk, teenagers, sexual and reproductive health, creative sexual development

## Abstract

It has been over a quarter of a century since the sexual reproductive health of young people came under the spotlight. The upsurge in human immunodeficiency virus (HIV) infections spurred on an era of intense development of services and strategies to ensure people's reproductive health rights were attainable, including the right to choose when to fall pregnant and have a baby. The statistics on teenage pregnancy are more than just numbers, but a represent stark reality for some girls in South African schools. Given that pregnancy in the teenage years is largely unintentional, prevention strategies need to extend beyond addressing risky sexual behavior; gaining deeper insights into teenagers' experiences and the events leading up to pregnancy would serve to better inform pregnancy prevention programs. This study explored the experiences of teenage mothers and pregnant teenagers, with the objective of acquiring a broader understanding of alternative approaches to preventing unintended pregnancy. A qualitative study was conducted in Ekurhuleni's township in the east of Johannesburg, South Africa. Fifteen narrative interviews with girls aged 13–19 years were conducted between July 2015 and July 2016, and were analyzed chronologically through narrative analysis. The findings revealed that participants who had engaged in socio-sexual and romantic relationships had no intention of falling pregnant and were familiar with existing strategies to prevent pregnancy. Social-sexual relationships were presented as an important aspect of their lives and demonstrated their ability to create spaces and opportunities to spend time with their social sexual partners and engage in sexual activity. Focusing on how teenage girls evaluate their sexual activity against the consequences of their actions is critical. However, sexual and reproductive health programs should refrain from representing young people's sexual behavior as a pathological condition, framing it instead as an integral component of creative sexual development. Programs should include relevant practical advice in relation to sexual engagement and be considered an extension of the State's existing Road to Health program.

## Introduction

Unintended teenage pregnancy is a challenge facing South Africa and various parts of the world, including developing and developed countries.

The high rate of teenage pregnancy, defined as occurring before the age of 19 (1), threatens the futures of many teenage girls in South Africa and Southern African Development Region (2). They risk serious health, socio-economic and educational challenges (3, 4), including the early termination of schooling due to policy, social norms or material conditions (5).

A World Health Organization (WHO) report indicates that the average global birth rate among 15–19 year-olds is 49 births per 1,000 girls. The highest figures are in sub-Saharan Africa, where rates as high as 299 per 1,000 have been recorded (1). The WHO report also reveals that adolescent pregnancy remains a major contributor to maternal and child mortality, and to the cycle of poverty and ill-health (6).

Over the past three decades, the rates of teenage pregnancy in South Africa have remained high, recurring pregnancies before completion of high school continue to be reported, and school drop-outs by grade 10, followed by pregnancy, remain common (7–10).

South African studies indicate that the rate of teenage pregnancy remains largely unchanged, despite efforts such as youth-friendly services and national adolescent friendly clinic initiatives introduced in South Africa in the late nineties (11). However, Branson and Byker (12) study indicates that if the teenagers stay closer to the friendly clinic there is some evidence of delayed childbearing, this however, evidence from one study. In June 2018, the Sowetan newspaper published an article with the headline: “1,000 schoolgirls fall pregnant in 1 year—grade 5 pupil youngest on list” (13). Although the MEC declined to disclose the learner's age, considering her grade, she probably would have been between 10 and 12 years old.

In some cultures, pregnancy and childbearing increases the probability of marriage. In developing countries, one in three women aged 20–24 married before the age of 18, a third of whom married before the age of 15 (5).

Research on teenage pregnancy reveals that coercion or rape of young girls is a frequent cause of unintended pregnancy (14, 15). A systemic review identified sociocultural, environmental, economic and individual factors to have an influence on adolescent pregnancy (16), found that other reasons young women fell pregnant were ignorance, curiosity, peer pressure (17) or fear of attending clinics that were hostile toward adolescents seeking contraceptives (18).

A large proportion of pregnancies in South Africa are reportedly unplanned, which is not only a public health concern but also a development concern because of its impact on the country's economy (19). When girls' schooling is interrupted, it means the country has access to fewer skilled female workers and entrepreneurs to contribute to its economic development. It also means more women who are dependent on social assistance programs for their survival because they have had children before they are economically independent.

Thus, far, the most frequently touted solution for preventing unintended teenage pregnancy is responsible sexual behavior. Biomedical approaches to teenage pregnancy focus on methods that contribute to the management of pregnancy outcomes, making recommendations for the prevention of teenage pregnancy. These include sexual health education, parent–child communication about sex, and setting up prevention programs aimed exclusively at young people (20). Review of 20 studies in both the high and low income countries found that there is lack of tools and indicators to measure effectiveness of youth friendly services (21).

Therefore, this study proposed exploring the viability of moving from a framework of “sexual risk” to one of “creative sexual development.” The framework has its origins in neuroscience, it attempts to explain young people cognitive capacity to engage in romantic sexual relationships (22). It explain how young people move from relationships free of sexual and romantic attraction and how these relationship are important for their social transition and that they derive satisfaction from these interactions. The important aspect is to pay attention to the young people's creativity in making spaces to derive satisfaction from social-sexual activities.

## Research Method

A qualitative research approach was selected because it allowed the researcher to perform an in-depth investigation with the object of the study (23). A narrative approach provided insights into the way human beings understood their experiences. It facilitated issues from the informants' perspective and did not attempt to explain or predict human behavior as emphasized by quantitative researchers (24). The narratives allowed the exploration of interviewees' perceptions against the context of their behavior and knowledge of pregnancy prevention.

### Sampling

This study was part of the larger study which employed a mixed method study, where young people participated in survey, focus group discussions and the narratives. The study was conducted in a township, east of Johannesburg which has 13 locations with varying characteristics in socio-economic status. Participants were recruited from public areas in the community such as clubs and sports fields, where presentations about the study were conducted. Participants who were interested contacted the recruiter and the researcher on any Friday afternoon or Saturday. The first participant in the narrative interview responded to recruitment message provided during the presentation at the Saturday study club. This paper focuses on the narrative aspect of the research. Purposive, convenience and snowball non-probability sampling strategies were employed to recruit teenage girls who had experienced an unintended pregnancy and who were able to identify other teenagers who had had a similar experience. In most cases they will know that there is a teenage mother in the next street but they did not know each by name, they knew each from meeting either at the clinic or school. There was no stringent inclusion or exclusion criteria; the prospective participants had to be pregnant, been pregnant, available and accessible for data collection. They should have been pregnant before they turned 19 years, and all 13–19 year olds were eligible. A total of 15 participants were recruitment and participated, two were pregnant and 13 had babies. The ages of participant was 15–19 years, the age at pregnancy was between 14 and 18 years.

### Data Collection

Data was collected over a period on 1 year, from June 2015 to July 2016 owing to the difficulty in recruiting teenage mothers and pregnant teenagers. The study employed narratives to collect and analyze data relating to teenagers' understanding of and behavior regarding the prevention of unintended pregnancy. Participants were asked to tell the story surrounding their pregnancy, prompted by the question: “Tell me your life story, any information related to your pregnancy, and event(s) leading to your pregnancy.” There was no research instrument, participant were asked to tell their story and there were no leading questions.

Each interview took place at a suitable time and in a setting chosen by the participant. While some participants chose to be interviewed in the comfort of their own homes, others preferred to have it take place in a house in the township that served as a data collection point. After obtaining consent, a single interview averaging 30–45 min was conducted with each participant. Narratives were audio-recorded, and saved onto a backed up password-protected laptop.

### Data Analysis

The narratives enabled the researcher to identify recurrent themes, attitudes, and ideas (25) relating to unintended teenage pregnancy. As it was important to identify the events that led up to the pregnancy and their consequences, the stories were rearranged chronologically (26), using Ritchie and Spencer's framework (27). The stories were presented as follows: prior to pregnancy, during pregnancy and post-delivery, which included their current state.

Through multiple readings of the transcripts, labeling, coding, recoding and analyzing data, the data was condensed into final concepts. The study coding and analysis was verified by an inter-coder, who is a researcher with expertise in qualitative research.

An audit trail of recordings, transcripts, notes and coding was established. Authenticity was maintained by accurately representing the participants' perceptions, and allowing each participant to direct the flow of the interview. Emphasis was placed on how participants perceived the events and not on the facts *per se*. Understanding how events are located in a social context, rather than reproducing the past exactly as it was, is the main determinant of validity.

### Ethical Considerations

The study was explained to the participants and written informed consent was obtained prior to the start of the interviews. Informed consent was obtained from parents of girls younger than 18 years (the age of majority in South Africa) (28). Participants younger than 18 provided assent while participants older than 18 provided consent. To maintain confidentiality, participants were assigned code numbers instead of using their names. They were assured of anonymity, and were informed that their names would not be referred to in reporting the findings or in any form of data sharing such as conference presentations. The University of the Witwatersrand Human Research Ethics Committee (Medical) approved the study (protocol clearance number M140945).

### Study Limitation

The initial plan was to interview both boys and girls, using all three strategies of sampling, to gain a broader perspective of participants' experiences, both in terms of being pregnant and impregnating someone. However, boys who had impregnated a girl were unwilling to participate.

## Findings

The main findings of the study confirmed the unintentionality of the pregnancy. The events leading up to the pregnancy were mainly the result of being in a relationship and having fun.

### Demographic Information

The demographics and pregnancy history of the 15 girls who participated in the study are described in Table 1. The mean age of the participants at the time of their interview was 18 years. All 15 participants were still in school, grades 10–12. Thirteen had already had their babies and two were still pregnant.

**Table 1:** Demographic composition of narrative participants, including relationships and sexual activity.

**Participant #**	**Participant**	**Partner**	**Age at beginning**	**Age first sexual**	**Age at start**	**Grade at**	**Current**
	**age**	**age**	**of relationship**	**encounter**	**of pregnancy**	**pregnancy**	**grade**
1	19	24	14	14	16	10	12
2	17	24	12	14	14 + 17	10	11
3	15	18	13	13	14	10	11
4	18	20	12	15	15	10	12
5	18	22	12	13	16	10	10
6	18	20	14	14	15	10	11
7	19	21	16	17	18	10	10
8	15	18	12	14	16	10	11
9	18	25	14	14	16	9	10
10	17	21	14	16	17	10	10
11	18	20	14	14	16	8	10
12	19	23	14	14	17	9	10
13	18	21	14	16	17	10	10
14	19	20	15	16	17	10	11
15	16	18	14	14	14	10	10

### Experiences and Activities Prior to Pregnancy

#### Social-Sexual Relationships

Relationships defined as dating (“ukujola” in the IsiZulu language) refer to social-sexual relationships (29) resulting from a young person making a conscious decision to be involved with another person, whether his/her peer, or an older or younger person. Some relationships were within the same age group and entailed an emotional connection. All participants interviewed had been or were currently in a heterosexual relationship. The age of their first social-sexual relationship ranged from 12 to 16, starting in grade 7 or grade 8.

The participants viewed being in a relationship as acceptable and not negative, but their views about having sexual encounters within these relationships varied. What was evident is that sexual encounters were part of these social-sexual relationships.

### Sexual Activity as Part of Dating, but Not Pregnancy

Sexual activity within this cohort occurred between the ages of 13 and 17, mean age 14. The first sexual encounter with the boy that made the participant pregnant occurred within the first 2 years of the relationship. Only one participant reported that her sexual encounter with her boyfriend was non-consensual.

The participants reported being aware that a sexual encounter could result in pregnancy, but either did not think about pregnancy or did not think they could become pregnant.

“*I never thought of being pregnant.”* (Participant 10, age 17, currently pregnant)

“*I did not know that I am pregnant, but I knew that when you have sex you will be pregnant*. (Participant 5, age 18, teenage mother).

### Pull Factors for Sexual Activity

The researcher observed from participants' stories that certain factors such as going out, drinking alcohol, or being in a relationship created opportunities for sexual interaction. They considered having a sexual relationship to be enjoyable, but acknowledged that such behaviors led to engaging in sexual intercourse. The spaces they created often included removing themselves from their parents' supervision or monitoring. This creativity demonstrate teenage girls ability to act and go against their parents' will in order to attain something that serve a fulfilling purpose for themselves. According to neurosciences teenage creativity increase the condition for romantic and sexual relationships which are prosocial rather than risk taking (22).

“*One of our friends would make fake newsletters stating that there is soccer first or Valentine ball at school. But parents also are ignorant; how come there can be a Valentine's ball in September?”* (Participant 3, age 15, teenage mother)

“*When I left home I never said I was going to jive; they were not going to permit me, so I will just say I am going to visit my friend. When we leave my friend's house, we will say we are going to visit at my house. That way we would be able to go*.” (Participant 13, age 18, teenage mother)

Some participants mentioned that they had had sexual intercourse but it was “by mistake.” Sex was preceded by physical contact such as hugging and kissing. The majority had been partying and drinking prior to the sexual encounter, and seemed not to worry about the consequences, provided they were having fun. They admitted to either being in a place where alcohol was consumed, or consuming alcohol themselves, and where sexual activity was part of the game.

They had sex, did not use condoms and did not worry or think about anything. The only aspect of their behavior that they regarded as negative was pregnancy, because it happened when least expected. As participants' stories of the events leading to pregnancy unfolded, it became apparent that their relationships and other activities in their lives had shaped their decision to engage in sexual activity.

### Perceptions and Knowledge About Pregnancy Prevention

Through their narratives, the participants indicated that they had not intended to fall pregnant. Some also stated that although they had clear knowledge about how pregnancy occurred, most did not take the necessary precautions.

“*Mm, no, I did not think; I was still very young. I don't want to lie. I did not think of them [pregnancy prevention], I am only thinking of them [pregnancy prevention] now that I have (Name of baby).”* (Participant 9, age 18, teenage mother)

The teenagers discovered in various ways that they were pregnant and at different stages of pregnancy, from 1 to 7 months.

### Post Pregnancy Regrets and Introspection

All participants felt strongly that their pregnancy was a “mistake” and were now taking precautions against pregnancy. Their regrets were informed by various situations in which they found themselves, such as failed relationships and timing which affected their schooling. Some regretted the financial strain to which they had subjected their families.

“*No, we did not plan, it's just something that happened …”* (Participant 4, age 18, teenage mother)

“*It makes me feel so bad. I feel like I am a bad person in that I should not have slept with him from the start.”* (Participant 11, age 18, teenage mother)

The participants' regrets were mostly linked to the impact of the pregnancy and child birth on their schooling and their plans for the future. All participants continued with their schooling during pregnancy, with some delaying their return after having their babies. Having a baby seemed to be a key motivator to study and have a better future, while also building a better future for the baby.

“*I would have passed and not failed. I would be finishing by now and next I go to school and complete my studies. Now I am behind.”* (Participant 13, 18, teenager mother)

“*I think that I should have finished school first, went to those universities, and worked and have a child when already married*.” (Participant 14, age 19, teenage mother)

## Discussion

The participants' stories demonstrate that they had the power to make decisions and create spaces and opportunities to be with their sexual partners. At the same time, those same stories demonstrate their failure to evaluate the outcome of sexual activity prior to pregnancy. The upshot was living with regrets, while using their experience of unintended pregnancy and the subsequent interruption in their schooling to motivate them to make better decisions going forward.

This study identified factors contributing to pregnancy among teenage girls such as dating, sexual activity and other activities precipitating sexual activity such as partying and drinking alcohol. This view is supported in a study by Arceo-Gomez and Campos-Vasquez (30) which states that pregnancy usually results when two people who are in love have a pleasurable experience.

This study also demonstrated that unintended teenage pregnancy disturbed the equilibrium in young people's lives, highlighting the importance of finding a way of working with teenagers to realize their right to choose when and how to have a baby.

Participants in this study had knowledge about pregnancy prevention and reproductive health, confirming research that information on the prevention of unintended pregnancy and prevention is available (31). However, some studies suggest that the messages received by teenagers may be inadequate, conflicting or overwhelming (32).

A group of researchers examined a sex education program in Scotland called SHARE (Sexual Health and Relationships Education), which educated teenagers aged 13–15 years using small groups, pamphlets on sexual health, sexual negotiation skills and condom usage (33). The program, which aimed to reduce unwanted pregnancies and unsafe sex, and improve the quality of relationships, was found to have increased participants' knowledge of sexual health. However, it did not reduce conception or termination of pregnancies when compared with other sex education programs, which the researchers argued would only be achieved by addressing socio-economic inequalities (33). In addition, Wight et al. (34) found that this program did not reduce sexual risk-taking in adolescents. Some authors have alluded to the inability of teenagers to apply pregnancy prevention to sexual and reproductive health programs that failed to acknowledge that young people constructed their own meaning about their sexual selves (35).

Construed as a reality in their own situation (36), study participants viewed dating, sexual activity and teenage pregnancy as “issues” and not “problems.” Several authors have expressed concern that the focus on sexual danger has hidden under a shroud of secrecy the expression of love, desire and romance, and the thrills that comprise sexual relations (37, 38). The concept of “risky” sexual behavior tends to problematize the sexual behavior of teenagers, resulting in teenage sexuality being labeled a “pathological condition” (39). A 2016 study found that during puberty, young people transitioned from a relationship that was free from sexual attraction to a context in which sexual and romantic attraction became their highest priority (22). The findings highlighted the importance of focusing on how teenagers consider and measure the outcome of their social-sexual behaviors, with minimal condemnation of the behavior itself. Policy on sexual and reproductive health rights of young people draws on particular models but tends to disregard the reality of power and resourcefulness that exists within this group of the population.

Jones (40) recognized teenagers as individuals who possessed the power to think, act, experience and make choices. The participants displayed a strong flair for creating spaces and evading adult supervision to be alone with their social-sexual partners. They demonstrated their resourcefulness in creating spaces to be with their social-sexual partners, such as fabricating newsletters about school events, or leading their parents to believe they were studying with girlfriends so that they could attend parties where they would be unsupervised.

The sexual behavior of young people is shaped by their social and sexual relationships. This study highlighted the need to acknowledge that engaging in sex is not just a simple behavior, but also a social relationship. It is an important aspect of growth and development within a child's social environment (41), which comprises their home, community, peers and school, all of which have a direct and indirect influence on a young person's beliefs and behavior.

The participants' regrets regarding their unintended pregnancies were generally linked either to desertion by their boyfriend or interruption to their schooling. All but one did not regret engaging in sexual activity as such. Despite citing it as a reason for regretting their unintended pregnancy, interruption to schooling was minimal for all participants. This could be attributed to the school's pregnancy policy and educators' implementation of national policy by allowing girls to continue with their schooling during pregnancy and allowing them to return soon after having their baby.

A 2016 study by Gill et al. found that girls performed academically well following their pregnancy, suggesting that giving girls an opportunity to return to school rather than dropping out during pregnancy may positively influence their motivation to improve themselves. This was confirmed by the participants in this study, who demonstrated their eagerness to make a better future for themselves and their children.

## Conclusion

The research concluded that while teenagers were aware of their sexuality and were knowledgeable about the means to prevent pregnancy, many did not actively try to prevent pregnancy. This study's findings suggest that a message can be powerful, provided it is not positioned in a manner that violates people's beliefs or tries to prohibit them from doing something they are not willing to stop. The researcher recommended accommodating young people's wish to continue doing what they could not be stopped from doing, but to exercise caution.

In this study, the researcher undertook to move beyond a ‘sexual risk framework' (22) and arrived at the conclusion that adopting a ‘creative sexual developmental framework' would be far more effective. This framework would be used to understand how young people's discourse around sex and pregnancy is shaped, and how culture and beliefs influenced the development of their discourse which, in (42) words creativity is a desired trait. Drawing on the creativity and resourcefulness demonstrated by these young people, this framework could be used to encourage them to be equally creative in preventing pregnancy. It would incorporate applying the knowledge they have about pregnancy prevention and giving careful consideration to the timing and consequences of pregnancy. The road to health should not be limited to vaccination of younger children or young people attending HIV management but should include all young people. The road to health provides for an opportunity of the service to engage with a young person in an individualized manner thus getting to attend to individual circumstance. As an already existing service for children before teenage years, this intervention can be explored as an ongoing mechanism to support cognitive and social development. The interactions can be used to capitalize on how to deal with social-sexual relationships in a manner that benefit these young women and enable them to control their fertility timing.

## Data Availability Statement

The raw data supporting the conclusions of this article will be made available by the authors, without unduereservation.

## Ethics Statement

The studies involving human participants were reviewed and approved by University of the Witwatersrand Human Research Ethics Committee (Medical). Written informed consent to participate in this study was provided by the participants' legal guardian/next of kin.

## Author Contributions

The author confirms being the sole contributor of this work and has approved it for publication.

## Funding

This research was supported by National Research Foundation of South Africa (Unique Grant No. 105687) and the Faculty of Humanities Research Promotion Grant at the University of the Witwatersrand, Johannesburg in the Republic of South Africa. Writing up of the article is supported through Chancellor Female Academic Leadership Fellowship (FALF).

## Author Disclaimer

The statements made and views expressed are solely the responsibility of the researcher.

## Conflict of Interest

The author declares that the research was conducted in the absence of any commercial or financial relationships that could be construed as a potential conflict of interest.

## Publisher's Note

All claims expressed in this article are solely those of the authors and do not necessarily represent those of their affiliated organizations, or those of the publisher, the editors and the reviewers. Any product that may be evaluated in this article, or claim that may be made by its manufacturer, is not guaranteed or endorsed by the publisher.
